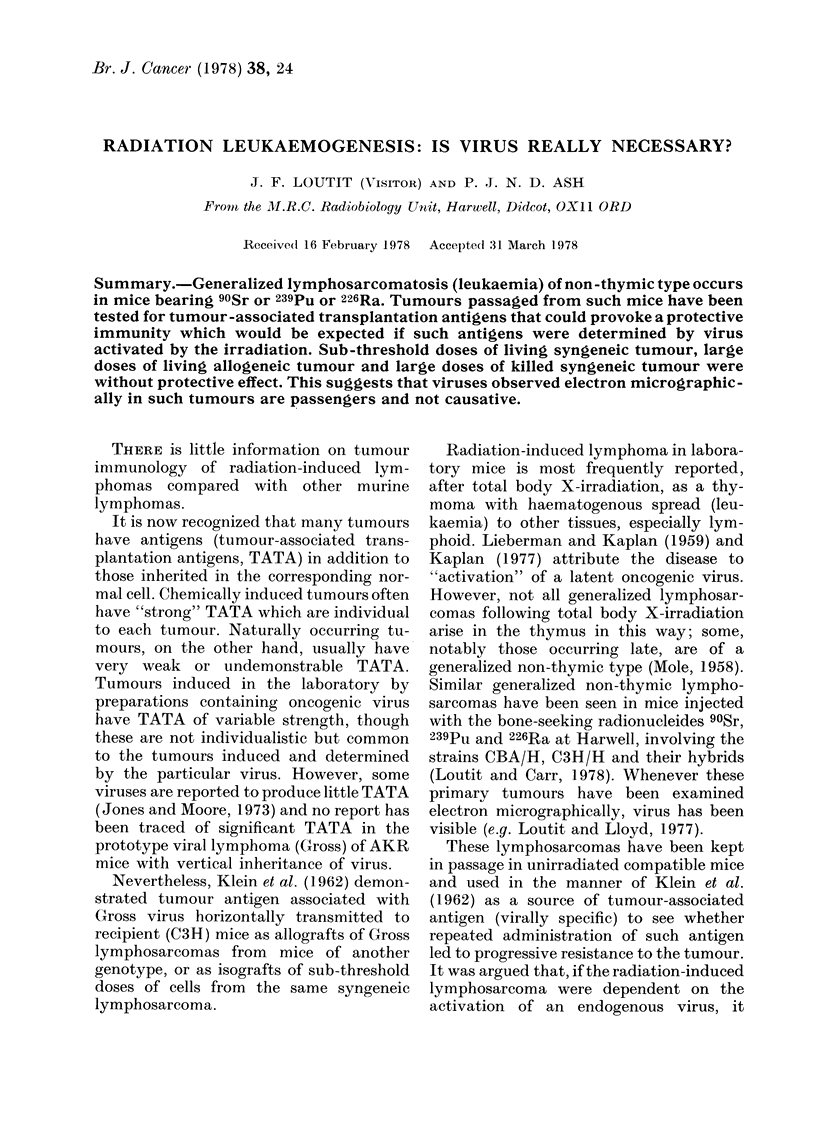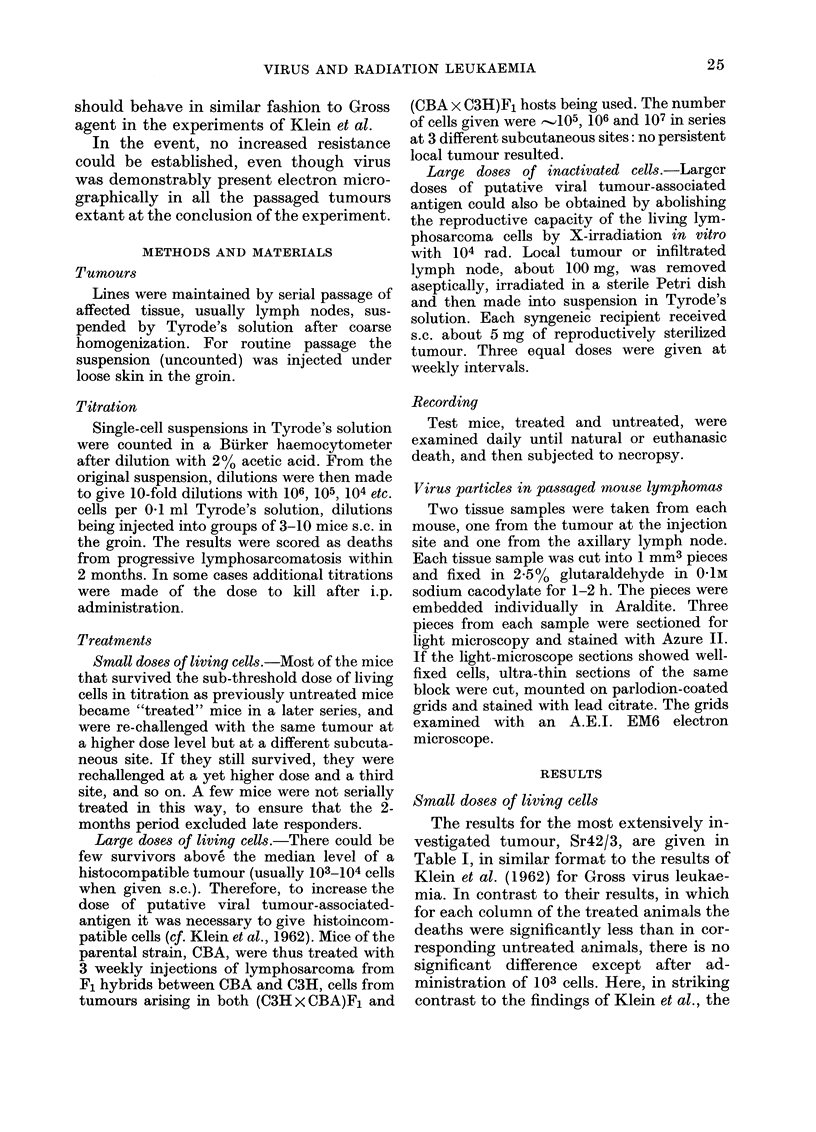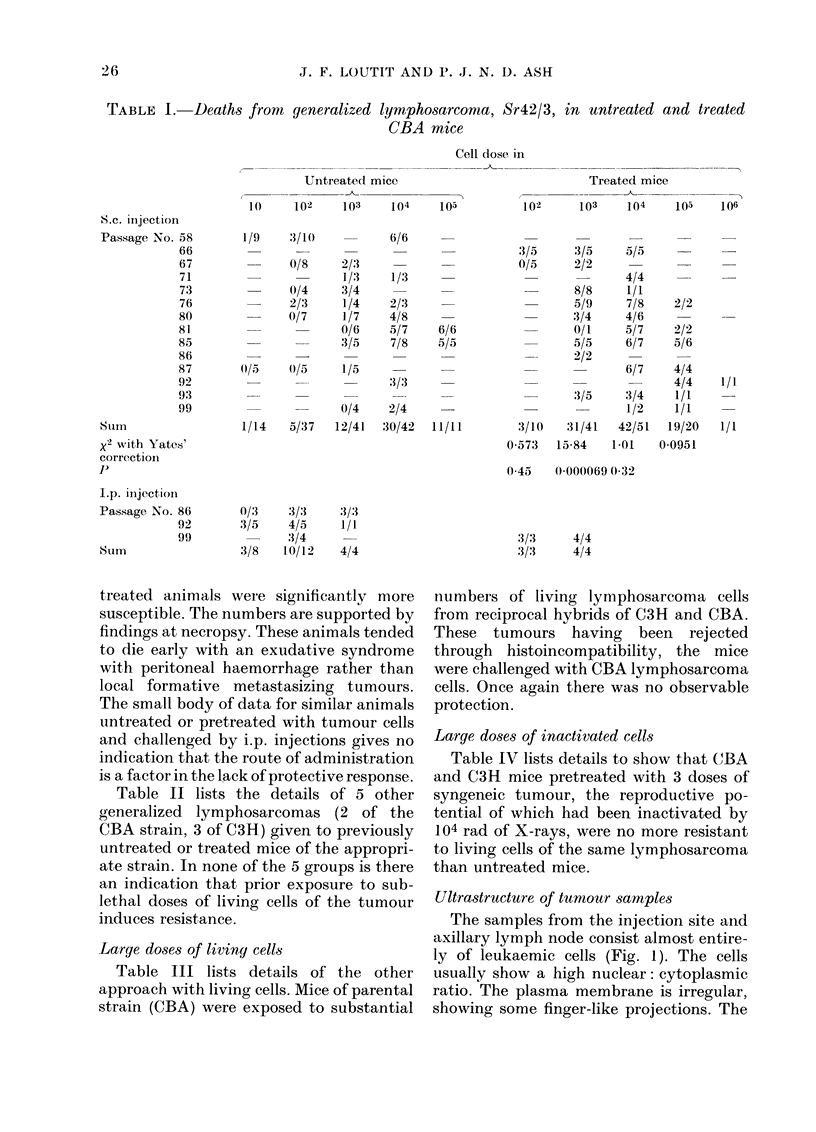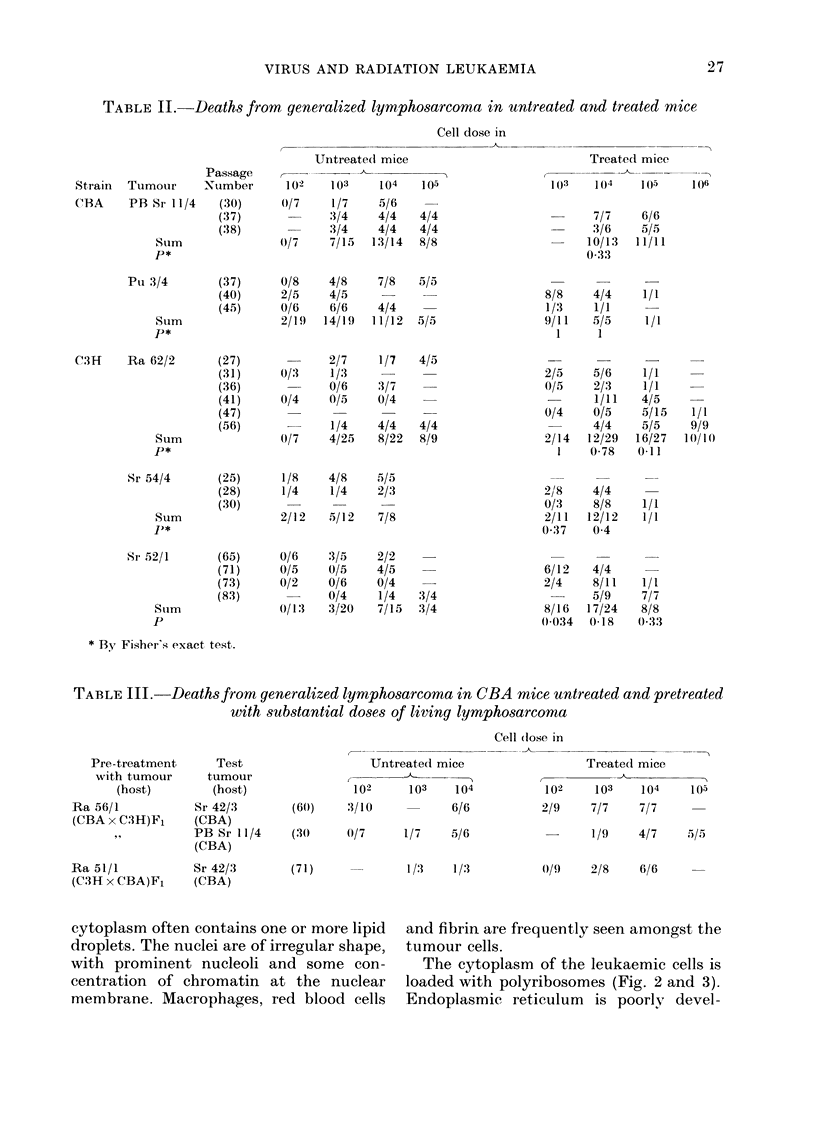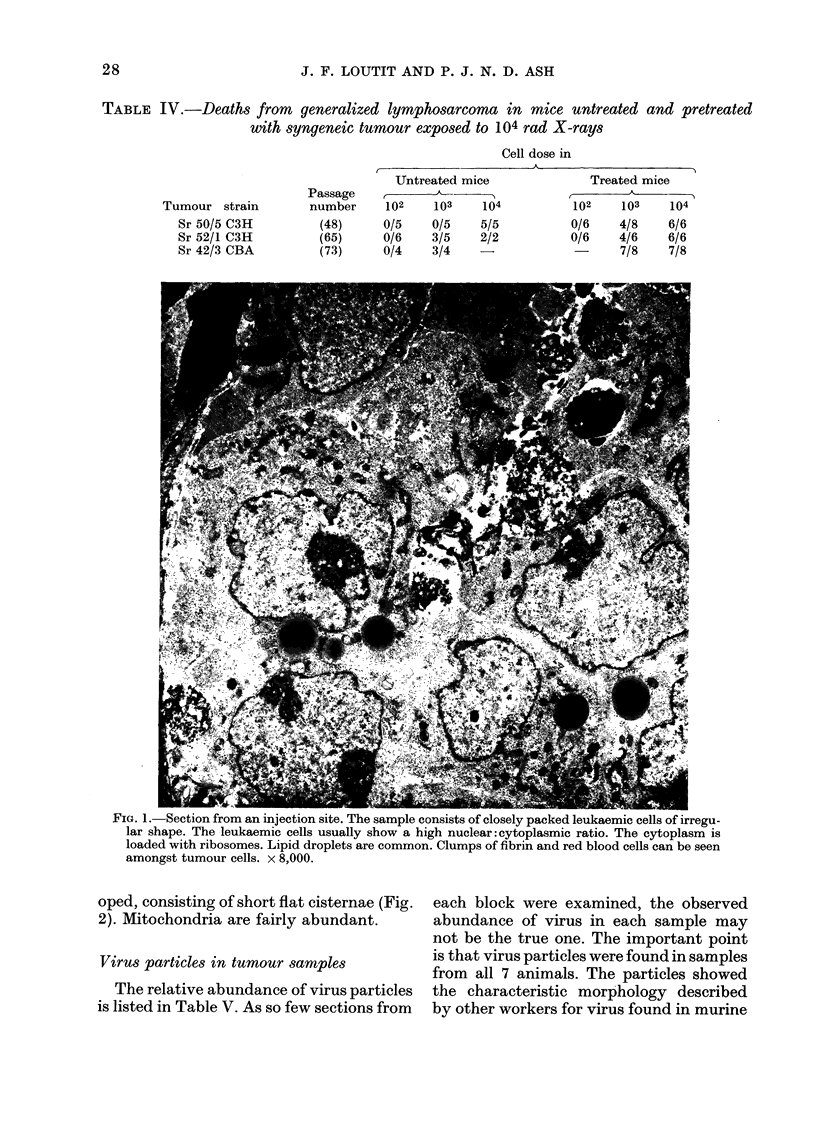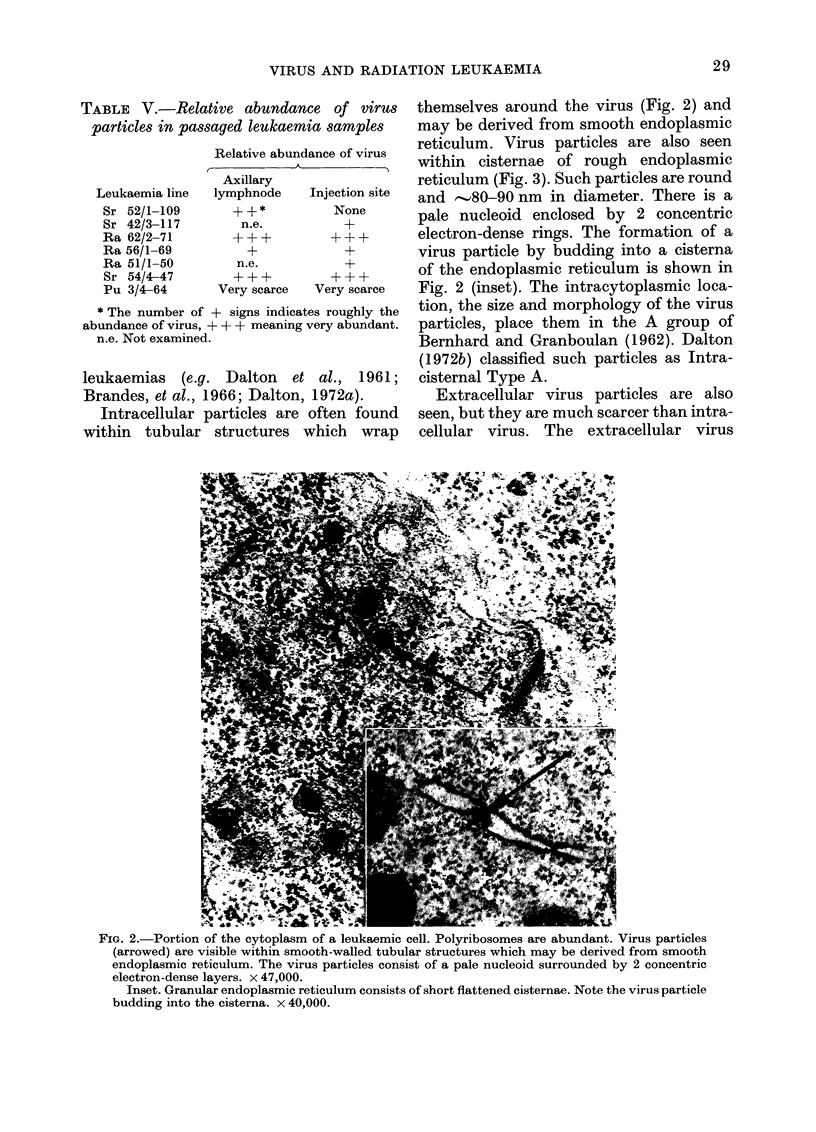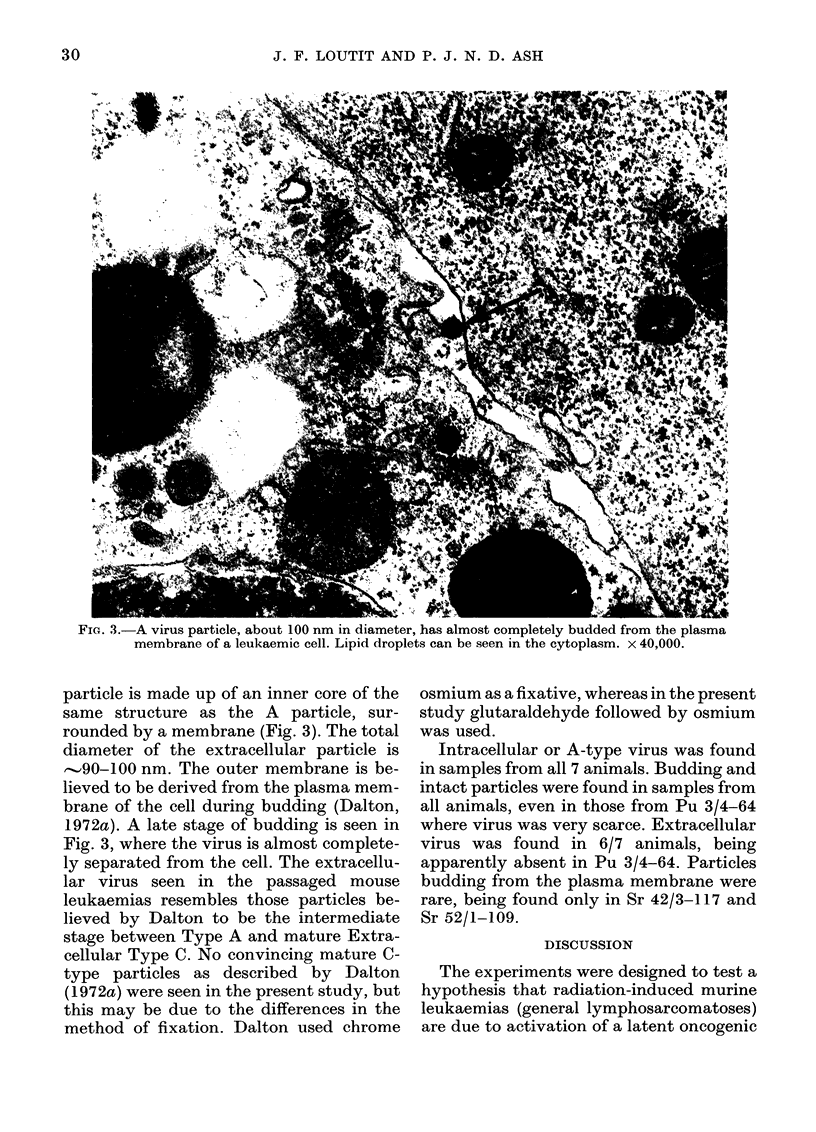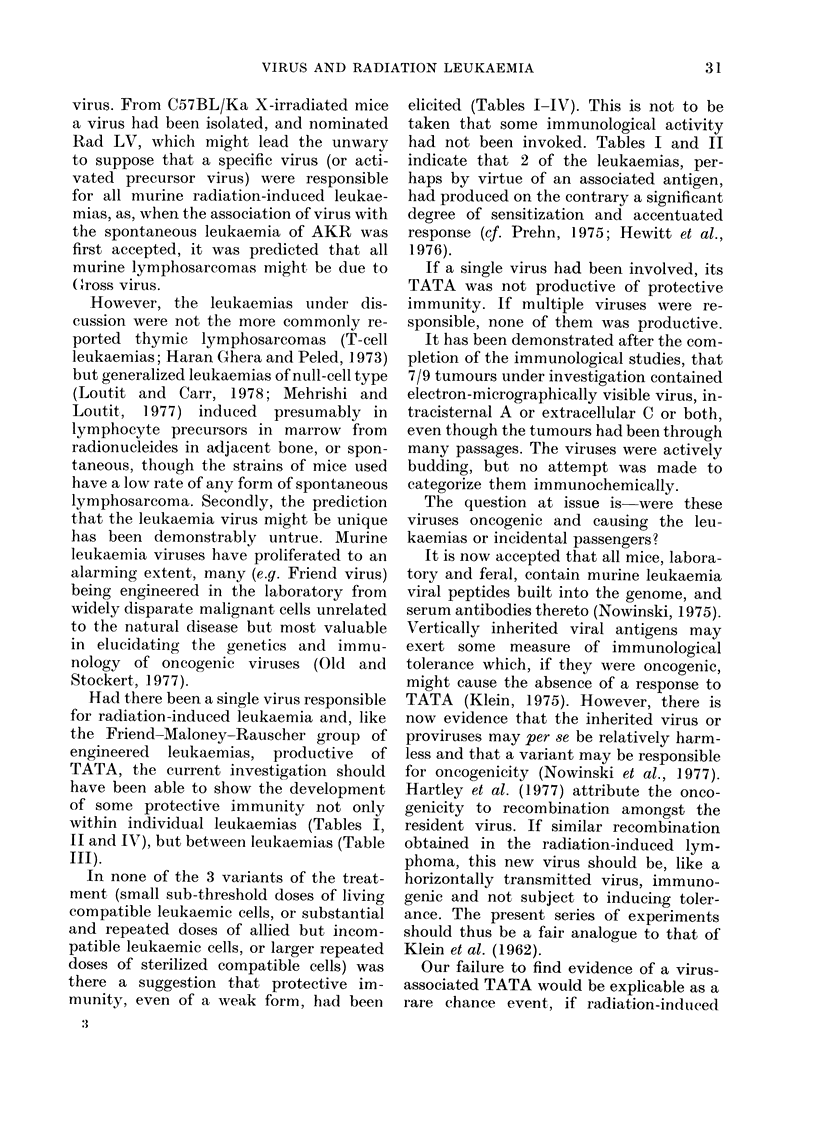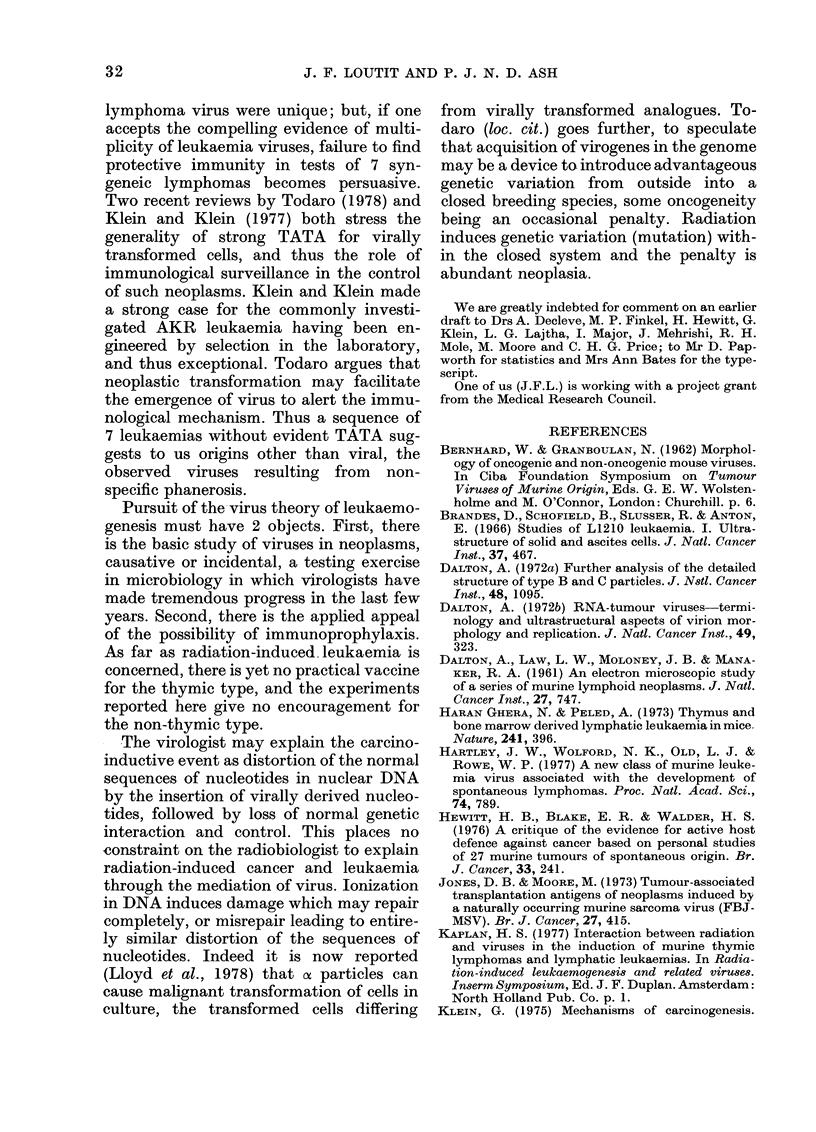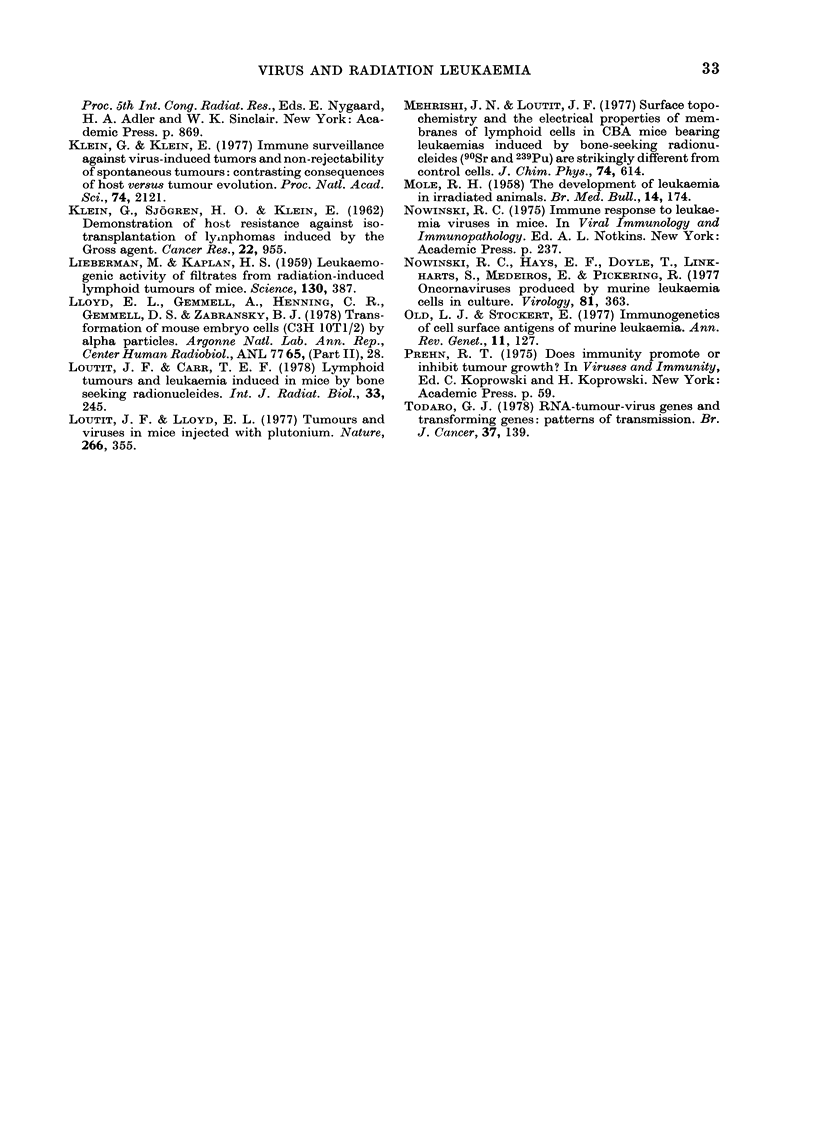# Radiation leukaemogenesis: is virus really necessary?

**DOI:** 10.1038/bjc.1978.160

**Published:** 1978-07

**Authors:** J. F. Loutit, P. J. Ash

## Abstract

**Images:**


*Br. J. Cancer* (1978) **38**, 24

### RADIATION LEUKAEMOGENESIS: IS VIRUS REALLY NECESSARY?

J. F. LOUTIT (VISITOR) AND P. J. N. D. ASH

*From the M.R.C. Radiobiology Unit, Harwell, Didcot, OX11 ORD*

Received 16 February 1978 Accepted 31 March 1978

**Summary.—Generalized lymphosarcomatosis (leukaemia) of non-thymic type occurs in mice bearing $^{90}$Sr or $^{239}$Pu or $^{226}$Ra. Tumours passaged from such mice have been tested for tumour-associated transplantation antigens that could provoke a protective immunity which would be expected if such antigens were determined by virus activated by the irradiation. Sub-threshold doses of living syngeneic tumour, large doses of living allogeneic tumour and large doses of killed syngeneic tumour were without protective effect. This suggests that viruses observed electron micrographically in such tumours are passengers and not causative.**

THERE is little information on tumour immunology of radiation-induced lymphomas compared with other murine lymphomas.

It is now recognized that many tumours have antigens (tumour-associated transplantation antigens, TATA) in addition to those inherited in the corresponding normal cell. Chemically induced tumours often have "strong" TATA which are individual to each tumour. Naturally occurring tumours, on the other hand, usually have very weak or undemonstrable TATA. Tumours induced in the laboratory by preparations containing oncogenic virus have TATA of variable strength, though these are not individualistic but common to the tumours induced and determined by the particular virus. However, some viruses are reported to produce little TATA (Jones and Moore, 1973) and no report has been traced of significant TATA in the prototype viral lymphoma (Gross) of AKR mice with vertical inheritance of virus.

Nevertheless, Klein *et al.* (1962) demonstrated tumour antigen associated with Gross virus horizontally transmitted to recipient (C3H) mice as allografts of Gross lymphosarcomas from mice of another genotype, or as isografts of sub-threshold doses of cells from the same syngeneic lymphosarcoma.

Radiation-induced lymphoma in laboratory mice is most frequently reported, after total body X-irradiation, as a thymoma with haematogenous spread (leukaemia) to other tissues, especially lymphoid. Lieberman and Kaplan (1959) and Kaplan (1977) attribute the disease to "activation" of a latent oncogenic virus. However, not all generalized lymphosarcomas following total body X-irradiation arise in the thymus in this way; some, notably those occurring late, are of a generalized non-thymic type (Mole, 1958). Similar generalized non-thymic lymphosarcomas have been seen in mice injected with the bone-seeking radionucleides $^{90}$Sr, $^{239}$Pu and $^{226}$Ra at Harwell, involving the strains CBA/H, C3H/H and their hybrids (Loutit and Carr, 1978). Whenever these primary tumours have been examined electron micrographically, virus has been visible (*e.g.* Loutit and Lloyd, 1977).

These lymphosarcomas have been kept in passage in unirradiated compatible mice and used in the manner of Klein *et al.* (1962) as a source of tumour-associated antigen (virally specific) to see whether repeated administration of such antigen led to progressive resistance to the tumour. It was argued that, if the radiation-induced lymphosarcoma were dependent on the activation of an endogenous virus, it should behave in similar fashion to Gross agent in the experiments of Klein *et al.*

In the event, no increased resistance could be established, even though virus was demonstrably present electron micrographically in all the passaged tumours extant at the conclusion of the experiment.

### METHODS AND MATERIALS

### *Tumours*

Lines were maintained by serial passage of affected tissue, usually lymph nodes, suspended by Tyrode's solution after coarse homogenization. For routine passage the suspension (uncounted) was injected under loose skin in the groin.

### *Titration*

Single-cell suspensions in Tyrode's solution were counted in a Bürker haemocytometer after dilution with 2% acetic acid. From the original suspension, dilutions were then made to give 10-fold dilutions with $10^6$, $10^5$, $10^4$ *etc.* cells per 0·1 ml Tyrode's solution, dilutions being injected into groups of 3–10 mice s.c. in the groin. The results were scored as deaths from progressive lymphosarcomatosis within 2 months. In some cases additional titrations were made of the dose to kill after i.p. administration.

### *Treatments*

*Small doses of living cells.*—Most of the mice that survived the sub-threshold dose of living cells in titration as previously untreated mice became "treated" mice in a later series, and were re-challenged with the same tumour at a higher dose level but at a different subcutaneous site. If they still survived, they were rechallenged at a yet higher dose and a third site, and so on. A few mice were not serially treated in this way, to ensure that the 2-months period excluded late responders.

*Large doses of living cells.*—There could be few survivors above the median level of a histocompatible tumour (usually $10^3$–$10^4$ cells when given s.c.). Therefore, to increase the dose of putative viral tumour-associated-antigen it was necessary to give histoincompatible cells (*cf.* Klein *et al.*, 1962). Mice of the parental strain, CBA, were thus treated with 3 weekly injections of lymphosarcoma from $F_1$ hybrids between CBA and C3H, cells from tumours arising in both (C3H × CBA)$F_1$ and (CBA × C3H)$F_1$ hosts being used. The number of cells given were ~$10^5$, $10^6$ and $10^7$ in series at 3 different subcutaneous sites: no persistent local tumour resulted.

*Large doses of inactivated cells.*—Larger doses of putative viral tumour-associated antigen could also be obtained by abolishing the reproductive capacity of the living lymphosarcoma cells by X-irradiation *in vitro* with $10^4$ rad. Local tumour or infiltrated lymph node, about 100 mg, was removed aseptically, irradiated in a sterile Petri dish and then made into suspension in Tyrode's solution. Each syngeneic recipient received s.c. about 5 mg of reproductively sterilized tumour. Three equal doses were given at weekly intervals.

### *Recording*

Test mice, treated and untreated, were examined daily until natural or euthanasic death, and then subjected to necropsy.

### *Virus particles in passaged mouse lymphomas*

Two tissue samples were taken from each mouse, one from the tumour at the injection site and one from the axillary lymph node. Each tissue sample was cut into 1 $mm^3$ pieces and fixed in 2·5% glutaraldehyde in 0·1M sodium cacodylate for 1–2 h. The pieces were embedded individually in Araldite. Three pieces from each sample were sectioned for light microscopy and stained with Azure II. If the light-microscope sections showed well-fixed cells, ultra-thin sections of the same block were cut, mounted on parlodion-coated grids and stained with lead citrate. The grids examined with an A.E.I. EM6 electron microscope.

### RESULTS

### *Small doses of living cells*

The results for the most extensively investigated tumour, Sr42/3, are given in Table I, in similar format to the results of Klein *et al.* (1962) for Gross virus leukaemia. In contrast to their results, in which for each column of the treated animals the deaths were significantly less than in corresponding untreated animals, there is no significant difference except after administration of $10^3$ cells. Here, in striking contrast to the findings of Klein *et al.*, the

TABLE I.—*Deaths from generalized lymphosarcoma, Sr42/3, in untreated and treated CBA mice*

| | Cell dose in | | | | | | | | | |
|---|---|---|---|---|---|---|---|---|---|---|
| | Untreated mice | | | | | Treated mice | | | | |
| | 10 | $10^2$ | $10^3$ | $10^4$ | $10^5$ | $10^2$ | $10^3$ | $10^4$ | $10^5$ | $10^6$ |
| S.c. injection | | | | | | | | | | |
| Passage No. 58 | 1/9 | 3/10 | — | 6/6 | — | — | — | — | — | — |
| 66 | — | — | — | — | — | 3/5 | 3/5 | 5/5 | — | — |
| 67 | — | 0/8 | 2/3 | — | — | 0/5 | 2/2 | — | — | — |
| 71 | — | — | 1/3 | 1/3 | — | — | — | 4/4 | — | — |
| 73 | — | 0/4 | 3/4 | — | — | — | 8/8 | 1/1 | | |
| 76 | — | 2/3 | 1/4 | 2/3 | — | — | 5/9 | 7/8 | 2/2 | |
| 80 | — | 0/7 | 1/7 | 4/8 | — | — | 3/4 | 4/6 | — | — |
| 81 | — | — | 0/6 | 5/7 | 6/6 | — | 0/1 | 5/7 | 2/2 | |
| 85 | — | — | 3/5 | 7/8 | 5/5 | — | 5/5 | 6/7 | 5/6 | |
| 86 | — | — | — | — | — | — | 2/2 | — | — | |
| 87 | 0/5 | 0/5 | 1/5 | — | — | — | — | 6/7 | 4/4 | |
| 92 | — | — | — | 3/3 | — | — | — | — | 4/4 | 1/1 |
| 93 | — | — | — | — | — | — | 3/5 | 3/4 | 1/1 | — |
| 99 | — | — | 0/4 | 2/4 | — | — | — | 1/2 | 1/1 | — |
| Sum | 1/14 | 5/37 | 12/41 | 30/42 | 11/11 | 3/10 | 31/41 | 42/51 | 19/20 | 1/1 |
| $\chi^2$ with Yates' correction | | | | | | 0·573 | 15·84 | 1·01 | 0·0951 | |
| *P* | | | | | | 0·45 | 0·000069 | 0·32 | | |
| I.p. injection | | | | | | | | | | |
| Passage No. 86 | 0/3 | 3/3 | 3/3 | | | | | | | |
| 92 | 3/5 | 4/5 | 1/1 | | | | | | | |
| 99 | — | 3/4 | — | | | 3/3 | 4/4 | | | |
| Sum | 3/8 | 10/12 | 4/4 | | | 3/3 | 4/4 | | | |

treated animals were significantly more susceptible. The numbers are supported by findings at necropsy. These animals tended to die early with an exudative syndrome with peritoneal haemorrhage rather than local formative metastasizing tumours. The small body of data for similar animals untreated or pretreated with tumour cells and challenged by i.p. injections gives no indication that the route of administration is a factor in the lack of protective response.

Table II lists the details of 5 other generalized lymphosarcomas (2 of the CBA strain, 3 of C3H) given to previously untreated or treated mice of the appropriate strain. In none of the 5 groups is there an indication that prior exposure to sublethal doses of living cells of the tumour induces resistance.

*Large doses of living cells*

Table III lists details of the other approach with living cells. Mice of parental strain (CBA) were exposed to substantial numbers of living lymphosarcoma cells from reciprocal hybrids of C3H and CBA. These tumours having been rejected through histoincompatibility, the mice were challenged with CBA lymphosarcoma cells. Once again there was no observable protection.

*Large doses of inactivated cells*

Table IV lists details to show that CBA and C3H mice pretreated with 3 doses of syngeneic tumour, the reproductive potential of which had been inactivated by $10^4$ rad of X-rays, were no more resistant to living cells of the same lymphosarcoma than untreated mice.

*Ultrastructure of tumour samples*

The samples from the injection site and axillary lymph node consist almost entirely of leukaemic cells (Fig. 1). The cells usually show a high nuclear : cytoplasmic ratio. The plasma membrane is irregular, showing some finger-like projections. The

TABLE II.—*Deaths from generalized lymphosarcoma in untreated and treated mice*

| | | | Cell dose in | | | | | | | |
|---|---|---|---|---|---|---|---|---|---|---|
| | | | Untreated mice | | | | Treated mice | | | |
| Strain | Tumour | Passage Number | $10^2$ | $10^3$ | $10^4$ | $10^5$ | $10^3$ | $10^4$ | $10^5$ | $10^6$ |
| CBA | PB Sr 11/4 | (30) | 0/7 | 1/7 | 5/6 | — | | | | |
| | | (37) | — | 3/4 | 4/4 | 4/4 | — | 7/7 | 6/6 | |
| | | (38) | — | 3/4 | 4/4 | 4/4 | — | 3/6 | 5/5 | |
| | Sum | | 0/7 | 7/15 | 13/14 | 8/8 | — | 10/13 | 11/11 | |
| | *P** | | | | | | | 0·33 | | |
| | Pu 3/4 | (37) | 0/8 | 4/8 | 7/8 | 5/5 | — | — | — | |
| | | (40) | 2/5 | 4/5 | — | — | 8/8 | 4/4 | 1/1 | |
| | | (45) | 0/6 | 6/6 | 4/4 | — | 1/3 | 1/1 | — | |
| | Sum | | 2/19 | 14/19 | 11/12 | 5/5 | 9/11 | 5/5 | 1/1 | |
| | *P** | | | | | | 1 | 1 | | |
| C3H | Ra 62/2 | (27) | — | 2/7 | 1/7 | 4/5 | — | — | — | — |
| | | (31) | 0/3 | 1/3 | — | — | 2/5 | 5/6 | 1/1 | — |
| | | (36) | — | 0/6 | 3/7 | — | 0/5 | 2/3 | 1/1 | — |
| | | (41) | 0/4 | 0/5 | 0/4 | — | — | 1/11 | 4/5 | — |
| | | (47) | — | — | — | — | 0/4 | 0/5 | 5/15 | 1/1 |
| | | (56) | — | 1/4 | 4/4 | 4/4 | — | 4/4 | 5/5 | 9/9 |
| | Sum | | 0/7 | 4/25 | 8/22 | 8/9 | 2/14 | 12/29 | 16/27 | 10/10 |
| | *P** | | | | | | 1 | 0·78 | 0·11 | |
| | Sr 54/4 | (25) | 1/8 | 4/8 | 5/5 | | — | — | — | |
| | | (28) | 1/4 | 1/4 | 2/3 | | 2/8 | 4/4 | — | |
| | | (30) | — | — | — | | 0/3 | 8/8 | 1/1 | |
| | Sum | | 2/12 | 5/12 | 7/8 | | 2/11 | 12/12 | 1/1 | |
| | *P** | | | | | | 0·37 | 0·4 | | |
| | Sr 52/1 | (65) | 0/6 | 3/5 | 2/2 | — | — | — | — | |
| | | (71) | 0/5 | 0/5 | 4/5 | — | 6/12 | 4/4 | — | |
| | | (73) | 0/2 | 0/6 | 0/4 | — | 2/4 | 8/11 | 1/1 | |
| | | (83) | — | 0/4 | 1/4 | 3/4 | — | 5/9 | 7/7 | |
| | Sum | | 0/13 | 3/20 | 7/15 | 3/4 | 8/16 | 17/24 | 8/8 | |
| | *P* | | | | | | 0·034 | 0·18 | 0·33 | |

* By Fisher's exact test.

TABLE III.—*Deaths from generalized lymphosarcoma in CBA mice untreated and pretreated with substantial doses of living lymphosarcoma*

| | | | Cell dose in | | | | | | |
|---|---|---|---|---|---|---|---|---|---|
| | | | Untreated mice | | | Treated mice | | | |
| Pre-treatment with tumour (host) | Test tumour (host) | | $10^2$ | $10^3$ | $10^4$ | $10^2$ | $10^3$ | $10^4$ | $10^5$ |
| Ra 56/1 (CBA × C3H)F1 | Sr 42/3 (CBA) | (60) | 3/10 | — | 6/6 | 2/9 | 7/7 | 7/7 | — |
| ,, | PB Sr 11/4 (CBA) | (30 | 0/7 | 1/7 | 5/6 | — | 1/9 | 4/7 | 5/5 |
| Ra 51/1 (C3H × CBA)F1 | Sr 42/3 (CBA) | (71) | — | 1/3 | 1/3 | 0/9 | 2/8 | 6/6 | — |

cytoplasm often contains one or more lipid droplets. The nuclei are of irregular shape, with prominent nucleoli and some concentration of chromatin at the nuclear membrane. Macrophages, red blood cells and fibrin are frequently seen amongst the tumour cells.

The cytoplasm of the leukaemic cells is loaded with polyribosomes (Fig. 2 and 3). Endoplasmic reticulum is poorly devel-

TABLE IV.—*Deaths from generalized lymphosarcoma in mice untreated and pretreated with syngeneic tumour exposed to* $10^4$ *rad X-rays*

| Tumour strain | Passage number | Cell dose in: Untreated mice $10^2$ | Untreated mice $10^3$ | Untreated mice $10^4$ | Treated mice $10^2$ | Treated mice $10^3$ | Treated mice $10^4$ |
|---|---|---|---|---|---|---|---|
| Sr 50/5 C3H | (48) | 0/5 | 0/5 | 5/5 | 0/6 | 4/8 | 6/6 |
| Sr 52/1 C3H | (65) | 0/6 | 3/5 | 2/2 | 0/6 | 4/6 | 6/6 |
| Sr 42/3 CBA | (73) | 0/4 | 3/4 | — | — | 7/8 | 7/8 |

FIG. 1.—Section from an injection site. The sample consists of closely packed leukaemic cells of irregular shape. The leukaemic cells usually show a high nuclear:cytoplasmic ratio. The cytoplasm is loaded with ribosomes. Lipid droplets are common. Clumps of fibrin and red blood cells can be seen amongst tumour cells. × 8,000.

oped, consisting of short flat cisternae (Fig. 2). Mitochondria are fairly abundant.

*Virus particles in tumour samples*

The relative abundance of virus particles is listed in Table V. As so few sections from each block were examined, the observed abundance of virus in each sample may not be the true one. The important point is that virus particles were found in samples from all 7 animals. The particles showed the characteristic morphology described by other workers for virus found in murine

TABLE V.—*Relative abundance of virus particles in passaged leukaemia samples*

| | Relative abundance of virus | |
|---|---|---|
| Leukaemia line | Axillary lymphnode | Injection site |
| Sr 52/1–109 | ++* | None |
| Sr 42/3–117 | n.e. | + |
| Ra 62/2–71 | +++ | +++ |
| Ra 56/1–69 | + | + |
| Ra 51/1–50 | n.e. | + |
| Sr 54/4–47 | +++ | +++ |
| Pu 3/4–64 | Very scarce | Very scarce |

* The number of + signs indicates roughly the abundance of virus, +++ meaning very abundant. n.e. Not examined.

leukaemias (*e.g.* Dalton *et al.*, 1961; Brandes, *et al.*, 1966; Dalton, 1972*a*).

Intracellular particles are often found within tubular structures which wrap themselves around the virus (Fig. 2) and may be derived from smooth endoplasmic reticulum. Virus particles are also seen within cisternae of rough endoplasmic reticulum (Fig. 3). Such particles are round and ~80–90 nm in diameter. There is a pale nucleoid enclosed by 2 concentric electron-dense rings. The formation of a virus particle by budding into a cisterna of the endoplasmic reticulum is shown in Fig. 2 (inset). The intracytoplasmic location, the size and morphology of the virus particles, place them in the A group of Bernhard and Granboulan (1962). Dalton (1972*b*) classified such particles as Intracisternal Type A.

Extracellular virus particles are also seen, but they are much scarcer than intracellular virus. The extracellular virus

FIG. 2.—Portion of the cytoplasm of a leukaemic cell. Polyribosomes are abundant. Virus particles (arrowed) are visible within smooth-walled tubular structures which may be derived from smooth endoplasmic reticulum. The virus particles consist of a pale nucleoid surrounded by 2 concentric electron-dense layers. × 47,000.

Inset. Granular endoplasmic reticulum consists of short flattened cisternae. Note the virus particle budding into the cisterna. × 40,000.

FIG. 3.—A virus particle, about 100 nm in diameter, has almost completely budded from the plasma membrane of a leukaemic cell. Lipid droplets can be seen in the cytoplasm. ×40,000.

particle is made up of an inner core of the same structure as the A particle, surrounded by a membrane (Fig. 3). The total diameter of the extracellular particle is ~90–100 nm. The outer membrane is believed to be derived from the plasma membrane of the cell during budding (Dalton, 1972*a*). A late stage of budding is seen in Fig. 3, where the virus is almost completely separated from the cell. The extracellular virus seen in the passaged mouse leukaemias resembles those particles believed by Dalton to be the intermediate stage between Type A and mature Extracellular Type C. No convincing mature C-type particles as described by Dalton (1972*a*) were seen in the present study, but this may be due to the differences in the method of fixation. Dalton used chrome osmium as a fixative, whereas in the present study glutaraldehyde followed by osmium was used.

Intracellular or A-type virus was found in samples from all 7 animals. Budding and intact particles were found in samples from all animals, even in those from Pu 3/4–64 where virus was very scarce. Extracellular virus was found in 6/7 animals, being apparently absent in Pu 3/4–64. Particles budding from the plasma membrane were rare, being found only in Sr 42/3–117 and Sr 52/1–109.

### DISCUSSION

The experiments were designed to test a hypothesis that radiation-induced murine leukaemias (general lymphosarcomatoses) are due to activation of a latent oncogenic virus. From C57BL/Ka X-irradiated mice a virus had been isolated, and nominated Rad LV, which might lead the unwary to suppose that a specific virus (or activated precursor virus) were responsible for all murine radiation-induced leukaemias, as, when the association of virus with the spontaneous leukaemia of AKR was first accepted, it was predicted that all murine lymphosarcomas might be due to Gross virus.

However, the leukaemias under discussion were not the more commonly reported thymic lymphosarcomas (T-cell leukaemias; Haran Ghera and Peled, 1973) but generalized leukaemias of null-cell type (Loutit and Carr, 1978; Mehrishi and Loutit, 1977) induced presumably in lymphocyte precursors in marrow from radionucleides in adjacent bone, or spontaneous, though the strains of mice used have a low rate of any form of spontaneous lymphosarcoma. Secondly, the prediction that the leukaemia virus might be unique has been demonstrably untrue. Murine leukaemia viruses have proliferated to an alarming extent, many (*e.g.* Friend virus) being engineered in the laboratory from widely disparate malignant cells unrelated to the natural disease but most valuable in elucidating the genetics and immunology of oncogenic viruses (Old and Stockert, 1977).

Had there been a single virus responsible for radiation-induced leukaemia and, like the Friend–Maloney–Rauscher group of engineered leukaemias, productive of TATA, the current investigation should have been able to show the development of some protective immunity not only within individual leukaemias (Tables I, II and IV), but between leukaemias (Table III).

In none of the 3 variants of the treatment (small sub-threshold doses of living compatible leukaemic cells, or substantial and repeated doses of allied but incompatible leukaemic cells, or larger repeated doses of sterilized compatible cells) was there a suggestion that protective immunity, even of a weak form, had been elicited (Tables I–IV). This is not to be taken that some immunological activity had not been invoked. Tables I and II indicate that 2 of the leukaemias, perhaps by virtue of an associated antigen, had produced on the contrary a significant degree of sensitization and accentuated response (*cf.* Prehn, 1975; Hewitt *et al.*, 1976).

If a single virus had been involved, its TATA was not productive of protective immunity. If multiple viruses were responsible, none of them was productive.

It has been demonstrated after the completion of the immunological studies, that 7/9 tumours under investigation contained electron-micrographically visible virus, intracisternal A or extracellular C or both, even though the tumours had been through many passages. The viruses were actively budding, but no attempt was made to categorize them immunochemically.

The question at issue is—were these viruses oncogenic and causing the leukaemias or incidental passengers?

It is now accepted that all mice, laboratory and feral, contain murine leukaemia viral peptides built into the genome, and serum antibodies thereto (Nowinski, 1975). Vertically inherited viral antigens may exert some measure of immunological tolerance which, if they were oncogenic, might cause the absence of a response to TATA (Klein, 1975). However, there is now evidence that the inherited virus or proviruses may *per se* be relatively harmless and that a variant may be responsible for oncogenicity (Nowinski *et al.*, 1977). Hartley *et al.* (1977) attribute the oncogenicity to recombination amongst the resident virus. If similar recombination obtained in the radiation-induced lymphoma, this new virus should be, like a horizontally transmitted virus, immunogenic and not subject to inducing tolerance. The present series of experiments should thus be a fair analogue to that of Klein *et al.* (1962).

Our failure to find evidence of a virus-associated TATA would be explicable as a rare chance event, if radiation-induced lymphoma virus were unique; but, if one accepts the compelling evidence of multiplicity of leukaemia viruses, failure to find protective immunity in tests of 7 syngeneic lymphomas becomes persuasive. Two recent reviews by Todaro (1978) and Klein and Klein (1977) both stress the generality of strong TATA for virally transformed cells, and thus the role of immunological surveillance in the control of such neoplasms. Klein and Klein made a strong case for the commonly investigated AKR leukaemia having been engineered by selection in the laboratory, and thus exceptional. Todaro argues that neoplastic transformation may facilitate the emergence of virus to alert the immunological mechanism. Thus a sequence of 7 leukaemias without evident TATA suggests to us origins other than viral, the observed viruses resulting from non-specific phanerosis.

Pursuit of the virus theory of leukaemogenesis must have 2 objects. First, there is the basic study of viruses in neoplasms, causative or incidental, a testing exercise in microbiology in which virologists have made tremendous progress in the last few years. Second, there is the applied appeal of the possibility of immunoprophylaxis. As far as radiation-induced leukaemia is concerned, there is yet no practical vaccine for the thymic type, and the experiments reported here give no encouragement for the non-thymic type.

The virologist may explain the carcino-inductive event as distortion of the normal sequences of nucleotides in nuclear DNA by the insertion of virally derived nucleotides, followed by loss of normal genetic interaction and control. This places no constraint on the radiobiologist to explain radiation-induced cancer and leukaemia through the mediation of virus. Ionization in DNA induces damage which may repair completely, or misrepair leading to entirely similar distortion of the sequences of nucleotides. Indeed it is now reported (Lloyd *et al.*, 1978) that α particles can cause malignant transformation of cells in culture, the transformed cells differing from virally transformed analogues. Todaro (*loc. cit.*) goes further, to speculate that acquisition of virogenes in the genome may be a device to introduce advantageous genetic variation from outside into a closed breeding species, some oncogeneity being an occasional penalty. Radiation induces genetic variation (mutation) within the closed system and the penalty is abundant neoplasia.

We are greatly indebted for comment on an earlier draft to Drs A. Decleve, M. P. Finkel, H. Hewitt, G. Klein, L. G. Lajtha, I. Major, J. Mehrishi, R. H. Mole, M. Moore and C. H. G. Price; to Mr D. Papworth for statistics and Mrs Ann Bates for the typescript.

One of us (J.F.L.) is working with a project grant from the Medical Research Council.

### REFERENCES

BERNHARD, W. & GRANBOULAN, N. (1962) Morphology of oncogenic and non-oncogenic mouse viruses. In Ciba Foundation Symposium on *Tumour Viruses of Murine Origin*, Eds. G. E. W. Wolstenholme and M. O'Connor, London: Churchill. p. 6.

BRANDES, D., SCHOFIELD, B., SLUSSER, R. & ANTON, E. (1966) Studies of L1210 leukaemia. I. Ultrastructure of solid and ascites cells. *J. Natl. Cancer Inst.*, **37**, 467.

DALTON, A. (1972*a*) Further analysis of the detailed structure of type B and C particles. *J. Nstl. Cancer Inst.*, **48**, 1095.

DALTON, A. (1972*b*) RNA-tumour viruses—terminology and ultrastructural aspects of virion morphology and replication. *J. Natl. Cancer Inst.*, **49**, 323.

DALTON, A., LAW, L. W., MOLONEY, J. B. & MANAKER, R. A. (1961) An electron microscopic study of a series of murine lymphoid neoplasms. *J. Natl. Cancer Inst.*, **27**, 747.

HARAN GHERA, N. & PELED, A. (1973) Thymus and bone marrow derived lymphatic leukaemia in mice. *Nature*, **241**, 396.

HARTLEY, J. W., WOLFORD, N. K., OLD, L. J. & ROWE, W. P. (1977) A new class of murine leukemia virus associated with the development of spontaneous lymphomas. *Proc. Natl. Acad. Sci.*, **74**, 789.

HEWITT, H. B., BLAKE, E. R. & WALDER, H. S. (1976) A critique of the evidence for active host defence against cancer based on personal studies of 27 murine tumours of spontaneous origin. *Br. J. Cancer*, **33**, 241.

JONES, D. B. & MOORE, M. (1973) Tumour-associated transplantation antigens of neoplasms induced by a naturally occurring murine sarcoma virus (FBJ-MSV). *Br. J. Cancer*, **27**, 415.

KAPLAN, H. S. (1977) Interaction between radiation and viruses in the induction of murine thymic lymphomas and lymphatic leukaemias. In *Radiation-induced leukaemogenesis and related viruses. Inserm Symposium*, Ed. J. F. Duplan. Amsterdam: North Holland Pub. Co. p. 1.

KLEIN, G. (1975) Mechanisms of carcinogenesis.

*Proc. 5th Int. Cong. Radiat. Res.*, Eds. E. Nygaard, H. A. Adler and W. K. Sinclair. New York: Academic Press. p. 869.

KLEIN, G. & KLEIN, E. (1977) Immune surveillance against virus-induced tumors and non-rejectability of spontaneous tumours: contrasting consequences of host *versus* tumour evolution. *Proc. Natl. Acad. Sci.*, **74**, 2121.

KLEIN, G., SJÖGREN, H. O. & KLEIN, E. (1962) Demonstration of host resistance against isotransplantation of lymphomas induced by the Gross agent. *Cancer Res.*, **22**, 955.

LIEBERMAN, M. & KAPLAN, H. S. (1959) Leukaemogenic activity of filtrates from radiation-induced lymphoid tumours of mice. *Science*, **130**, 387.

LLOYD, E. L., GEMMELL, A., HENNING, C. R., GEMMELL, D. S. & ZABRANSKY, B. J. (1978) Transformation of mouse embryo cells (C3H 10T1/2) by alpha particles. *Argonne Natl. Lab. Ann. Rep., Center Human Radiobiol.*, ANL 77 **65**, (Part II), 28.

LOUTIT, J. F. & CARR, T. E. F. (1978) Lymphoid tumours and leukaemia induced in mice by bone seeking radionucleides. *Int. J. Radiat. Biol.*, **33**, 245.

LOUTIT, J. F. & LLOYD, E. L. (1977) Tumours and viruses in mice injected with plutonium. *Nature*, **266**, 355.

MEHRISHI, J. N. & LOUTIT, J. F. (1977) Surface topochemistry and the electrical properties of membranes of lymphoid cells in CBA mice bearing leukaemias induced by bone-seeking radionucleides ($^{90}$Sr and $^{239}$Pu) are strikingly different from control cells. *J. Chim. Phys.*, **74**, 614.

MOLE, R. H. (1958) The development of leukaemia in irradiated animals. *Br. Med. Bull.*, **14**, 174.

NOWINSKI, R. C. (1975) Immune response to leukaemia viruses in mice. In *Viral Immunology and Immunopathology*. Ed. A. L. Notkins. New York: Academic Press. p. 237.

NOWINSKI, R. C., HAYS, E. F., DOYLE, T., LINKHARTS, S., MEDEIROS, E. & PICKERING, R. (1977 Oncornaviruses produced by murine leukaemia cells in culture. *Virology*, **81**, 363.

OLD, L. J. & STOCKERT, E. (1977) Immunogenetics of cell surface antigens of murine leukaemia. *Ann. Rev. Genet.*, **11**, 127.

PREHN, R. T. (1975) Does immunity promote or inhibit tumour growth? In *Viruses and Immunity*, Ed. C. Koprowski and H. Koprowski. New York: Academic Press. p. 59.

TODARO, G. J. (1978) RNA-tumour-virus genes and transforming genes: patterns of transmission. *Br. J. Cancer*, **37**, 139.